# Lymphoma-Associated Hemophagocytic Syndrome (LAHS) Masquerading as Severe Sepsis and Septic Shock

**DOI:** 10.7759/cureus.85850

**Published:** 2025-06-12

**Authors:** Shea Ruelas, Isuri S Weerasinghe, Samuel Woldeyohannes

**Affiliations:** 1 Internal Medicine, Baylor Scott & White Medical Center, Fort Worth, USA; 2 Neurology, University of Texas Southwestern Medical Center, Fort Worth, USA; 3 Internal Medicine, Texas Christian University, Fort Worth, USA

**Keywords:** hemophagocytic lymphohistiocytosis (hlh), multi-organ failure, percutaneous liver biopsy, sepsis and septic shock, t-cell lymphoma

## Abstract

Our case involves a patient who presented with abdominal pain, nausea, and vomiting and was found to have lactic acidosis, acute liver dysfunction, and renal failure. An intra-abdominal infection was initially suspected, but the infectious workup was negative, and he did not respond to antimicrobial therapy. He rapidly deteriorated despite aggressive supportive care. Further workup revealed evidence that his symptoms were being driven by hemophagocytic lymphohistiocytosis (HLH) syndrome of an unclear cause. A liver biopsy was obtained; however, before the pathology results, the patient developed internal hemorrhage due to severe coagulopathy, and he was not able to be resuscitated despite aggressive treatment. The liver biopsy revealed T-cell lymphoma of the liver, most likely a hepatosplenic subtype of lymphoma, which is quite rare compared to the incidence of B-cell lymphomas. Lymphomas can present in a diverse range of presentations and can be accompanied by co-syndromes such as HLH. HLH can cause serious morbidity and mortality through overactivation of the immune system, affecting multiple organ systems. This case demonstrates the need for a broad differential when approaching a patient with multi-organ failure, as malignancy can be the inciting condition, and effective treatment may depend on swift diagnosis.

## Introduction

Hemophagocytosis lymphohistiocytosis (HLH) is a rare immune activation syndrome where the overproduction of inflammatory cytokines and the inappropriate activation of cytotoxic lymphocytes and macrophages leads to significant morbidity and often eventual mortality. Roughly four patients in one million in the United States are diagnosed with HLH [1-5]. Common precipitating factors of HLH include malignancy, medications, autoimmune processes, infectious etiology, or an idiopathic cause. HLH due to malignancy (M-HLH) is the most common secondary form of HLH and can be the initial presentation with lymphoma being the most common malignancy; however, identification can be challenging, and thus this syndrome is often underdiagnosed [2]. HLH may be a disease mimic, initially appearing as an acute infection or septic shock. 

Our case focuses on malignancy, such as lymphoma, as the trigger for HLH. It is imperative for healthcare providers to recognize that lymphomas can be accompanied by confounding co-syndromes such as HLH. Patients with T-cell lymphoma hemophagocytic syndrome (LAHS) are more prone to disseminated intravascular coagulation (DIC) and coagulopathy compared to B-cell LAHS patients and typically have worse outcomes [6-7]. Our case report involves a patient who presented with lactic acidosis, acute liver dysfunction, and renal dysfunction. He had no history of fever, travel, or drug toxins that could explain his presentation. An intra-abdominal infection was initially suspected, but the infectious workup was negative, and he did not improve with antimicrobial therapy. He rapidly decompensated to multi-organ failure despite aggressive supportive care. Upon further workup, he met the criteria for HLH [4], but the inciting cause was not clear. A liver biopsy was obtained; however, before the pathology results, the patient developed internal hemorrhage due to severe coagulopathy, and he was not able to be resuscitated despite aggressive treatment. The liver biopsy later revealed T-cell infiltration of the liver, most likely representing a hepatosplenic T-cell lymphoma (HSCTL), a rare subtype of peripheral T-cell lymphoma (PTCL), which accounts for less than 5% of all PTCL cases [8,9]. This case demonstrates the need for a broad differential when approaching a patient with multi-organ failure, as malignancy can be the inciting condition, and effective treatment may depend on swift diagnosis.

## Case presentation

A 52-year-old gentleman with a past medical history significant for hypertension, tobacco abuse, and obesity initially presented to an outside hospital with two days of intractable nausea, vomiting, abdominal pain, and shortness of breath. No fever was reported, and he was afebrile on arrival. Initial presentation and lab work revealed acute renal failure and severe lactic acidosis. He was initiated on hemodialysis and started on broad-spectrum antibiotics. Initial CT imaging was significant for steatotic liver disease and upper abdominal lymphadenopathy within the porta hepatis region, as well as a non-occlusive portal vein thrombosis. HIDA scan showed hepatocellular dysfunction, and workup thus far did not support gallbladder dysfunction or bile duct obstruction. Given his symptoms, there was also concern for cholecystitis and mild pancreatitis. By the fifth day of admission, his lipase and liver function tests (LFTs) continued to rise, raising concern for fulminant liver failure. He was then transferred to a higher acuity facility, which is also an established liver transplant center.

**Figure 1:**
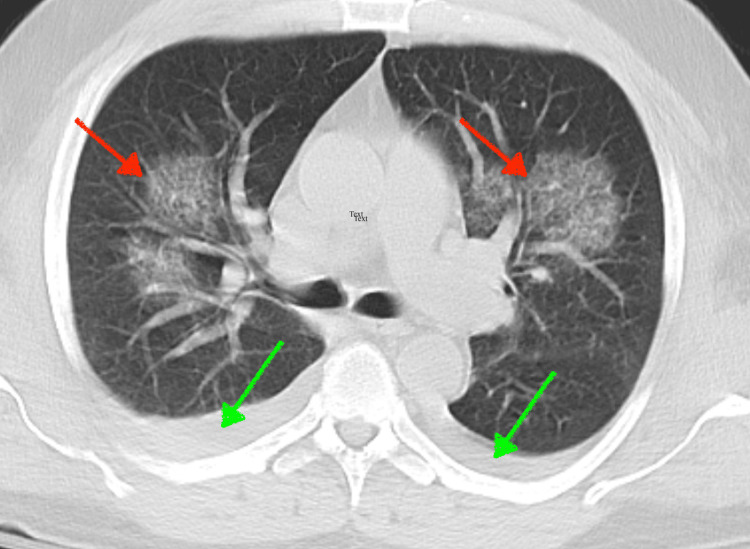
CT chest Lungs: Patchy groundglass opacities both upper lobes (red arrows). Small, bilateral pleural effusions with atelectatic change lung bases (green arrows). Enlarged subcarinal lymph gastroesophageal junction lymph nodes were identified (not shown).

**Figure 2:**
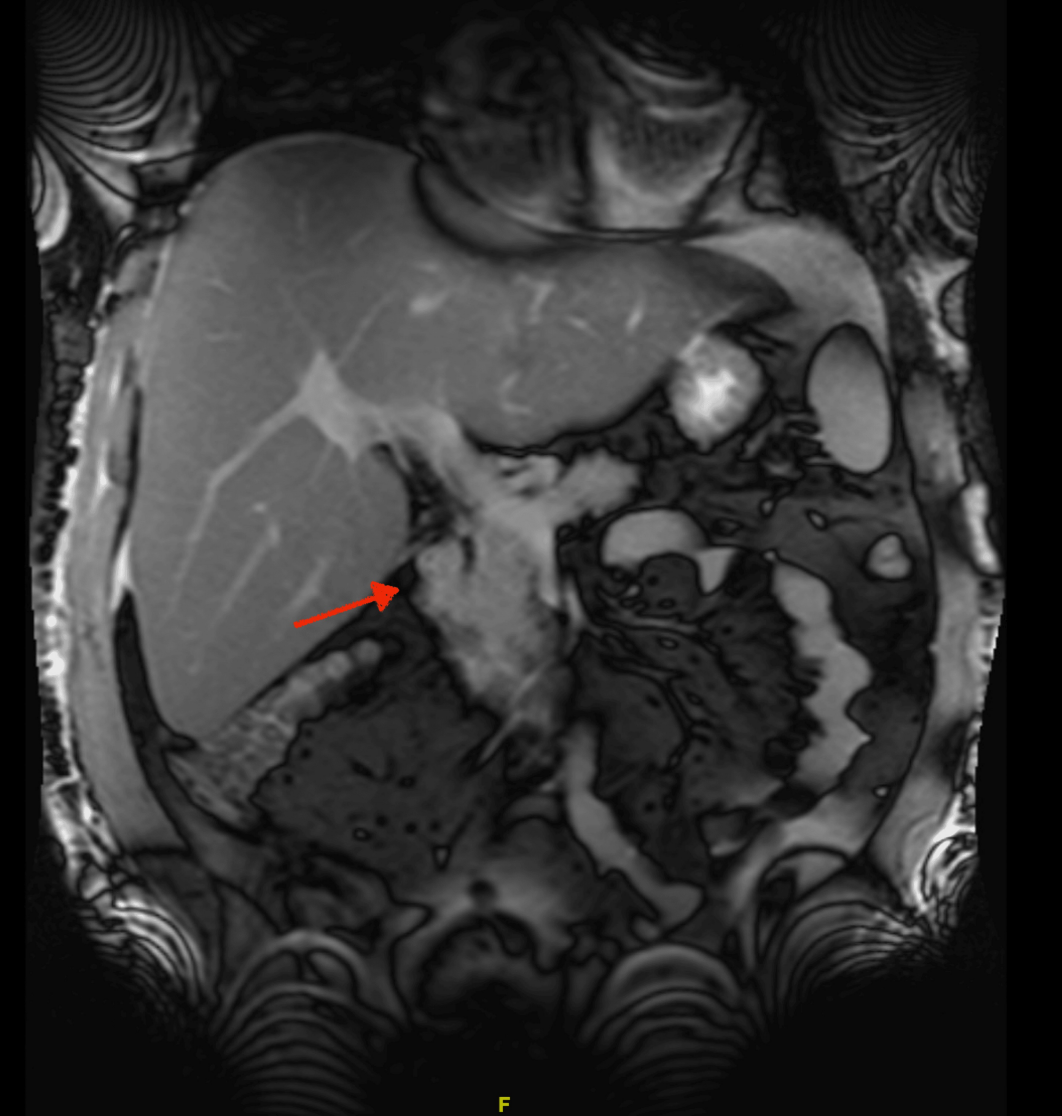
Magnetic resonance cholangiopancreatography (MRCP) of the abdomen Shows hepatic enlargement and enlarged portacaval lymph node approximating 1.1 cm short axis dimension (red arrow) with prominent lymph nodes porta hepatis.

Upon presentation to the higher acuity facility, the patient’s condition continued to deteriorate, requiring vasopressors and mechanical ventilation overnight. CT chest and abdomen/pelvis showed patchy groundglass opacities in both upper lobes, small bilateral pleural effusions with atelectatic changes in lung bases (Figure 1), fatty liver, and hepatosplenomegaly. Magnetic resonance cholangiopancreatography (MRCP) showed hepatosplenomegaly, gallbladder wall thickening, and an enlarged portacaval lymph node approximating 1.1 cm (Figure 2). Other imaging, such as US abdominal duplex, showed patent hepatic vasculature. Toxicology workup was negative for acetone, methanol, ethanol, acetaminophen, and salicylate toxicity. Patient’s antimicrobial coverage was broadened to piperacillin-tazobactam, doxycycline, and micafungin. Stress dose steroids were also added for refractory shock. Continuous renal replacement therapy was initiated due to worsening lactic acidosis and hypotension. Although his pH and LFTs showed some improvements with aggressive interventions, his lactic acid remained elevated, suspicious for Type B lactic acidosis caused by the Warburg effect (Table 1). Despite aggressive supportive care, the patient’s condition continued to decline.

**Table 1:** Laboratory trends

Lab trends	Day 1 (4/24/2024 outside hospital)	Day 2 (4/25/2023 outside hospital)	Day 3 (4/26/2023 outside hospital)	Day 4 (4/27/2023 outside hospital)	Day 5 (4/28/2023 liver transplant center)	Day 6 (4/29/2023 liver transplant center)	Day 7 (4/30/2023 liver transplant center	Day 8 (May 1 liver transplant center)	Day 9 (May 2 liver transplant center)	Reference range and units
Sodium	137	132	131	1341	131	132	136	134	135	136-145 meq/L
Potassium	5.8	3.8	3.7	3.6	3.4	4.1	3.9	4.1	4.2	3.6-5.0 meq/L
Chloride	96	95	93	87	85	84	79	75	67	98-107 meq/L
Carbon dioxide	7	15	15	15	14	18	19	13	25	21-32 meq/L
BUN	34	49	49	64	67	70	54	50	39	7-18 mg/dL
Creatinine	4.0	2.9	2.1	3.1	5.17	5.39	3.84	3.44	2.36	0.70-1.30 mg/dL
Bilirubin	0.6	0.8	1.9	2.8	4.0	4.6	6.5	8.9	7.8	0.2-1.0 mg/dL
Alkaline phosphatase	151	94	126	171	273	325	364	472	239	45-117 U/L
Aspartate transaminase	445	335	542	680	916	935	805	1167	786	15-37 U/L
Alanine transaminase	283	166	188	192	202	233	222	281	184	16-61 U/L
Lactic acid	12.86	14.29	15.19	19.74	17.8	20.1	22.8	31.7	34.5	0.9-1.7 mmol/L
Triglycerides			575			953		1542	814	0-150 mg/dL

On day eight of admission, he developed unstable atrial fibrillation with rapid ventricular response requiring cardioversion. Echocardiogram revealed normal left and right ventricular function with no significant valvular disease. Blood cultures, respiratory and stool cultures were negative, and he remained afebrile with worsening leukocytosis. Despite all interventions, his liver function continued to decline, his lactic acid continued to rise, and plasmapheresis was initiated to combat the elevated triglycerides. Clinical suspicion for HLH syndrome was high as the patient exhibited most of the diagnostic criteria, such as splenomegaly, hemoglobinemia, thrombocytopenia, hypertriglyceridemia, and increased ferritin. Other features of HLH were also evident, such as elevated LDH, elevated bilirubin, and hepatomegaly. At this point, four out of the eight HLH-2004 criteria were met; 5/8 are needed to make the diagnosis of HLH [7]. Soluble interleukin-2 receptor alpha (sCD25) levels were then collected to confirm the diagnosis of HLH. 

On day nine, the patient underwent a liver biopsy to try to determine the cause of liver dysfunction. Post-procedure, his ventilation requirements dramatically increased, and he developed worsening hypotension. Physical examination revealed that his abdomen was markedly distended and firm compared to being soft prior to the procedure. These findings were consistent with shock due to abdominal compartment syndrome. This was most likely due to DIC, confirmed by a hemostasis profile showing decreased platelets and fibrinogen levels and elevated prothrombin time. Massive transfusion protocol was initiated, and he was taken for emergency laparotomy. In the operating room, the patient was found to have massive hemoperitoneum, causing intestinal compression. Although no source of bleeding was identified, a non-expanding hematoma in the deep mesentery was visualized. Anesthesia reported acute oxygen desaturations and decreased breath sounds on the left side of the chest. An emergent thoracotomy was performed, and a chest tube was placed, with approximately 1 L of blood removed. A superficial vessel that was bleeding was also found and ligated. Bleeding from the abdomen was again noted in the deep mesentery. Despite efforts to control the bleeding from multiple sites, the patient continued to bleed internally and was noted to be bleeding from the nose and mouth as well. Over 70 units of blood products were administered intra-operatively, and the patient required multiple cardioversions due to supraventricular tachycardia. At this point, the family made the difficult decision to withdraw care and pursue comfort measures. An autopsy was not performed.

**Figure 3:**
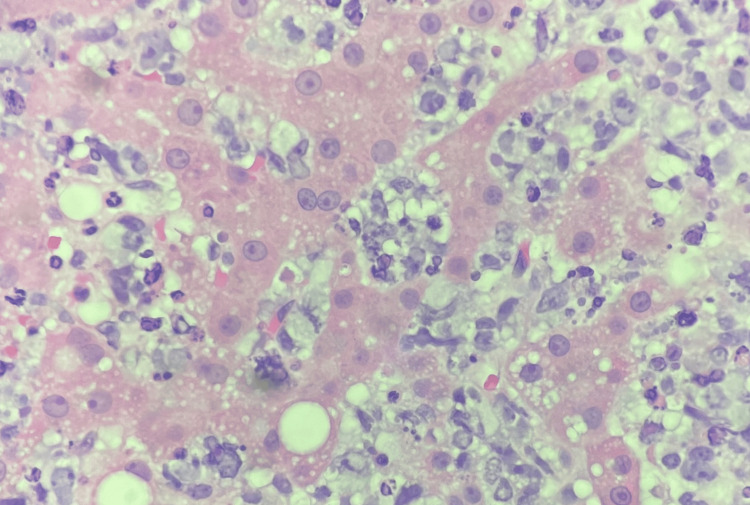
There is extensive infiltration of hepatic sinusoids by cells with small- to intermediate-size nuclei with exclusion of blood.

**Figure 4:**
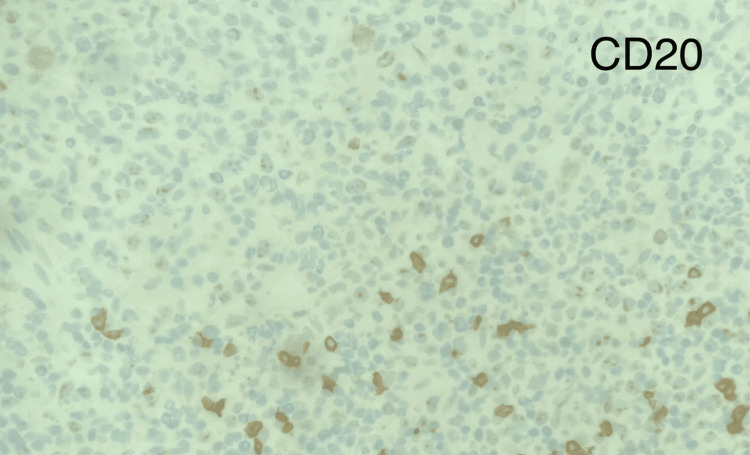
CD20 stain on liver biopsy demonstrates small amounts of B-cells present.

**Figure 5:**
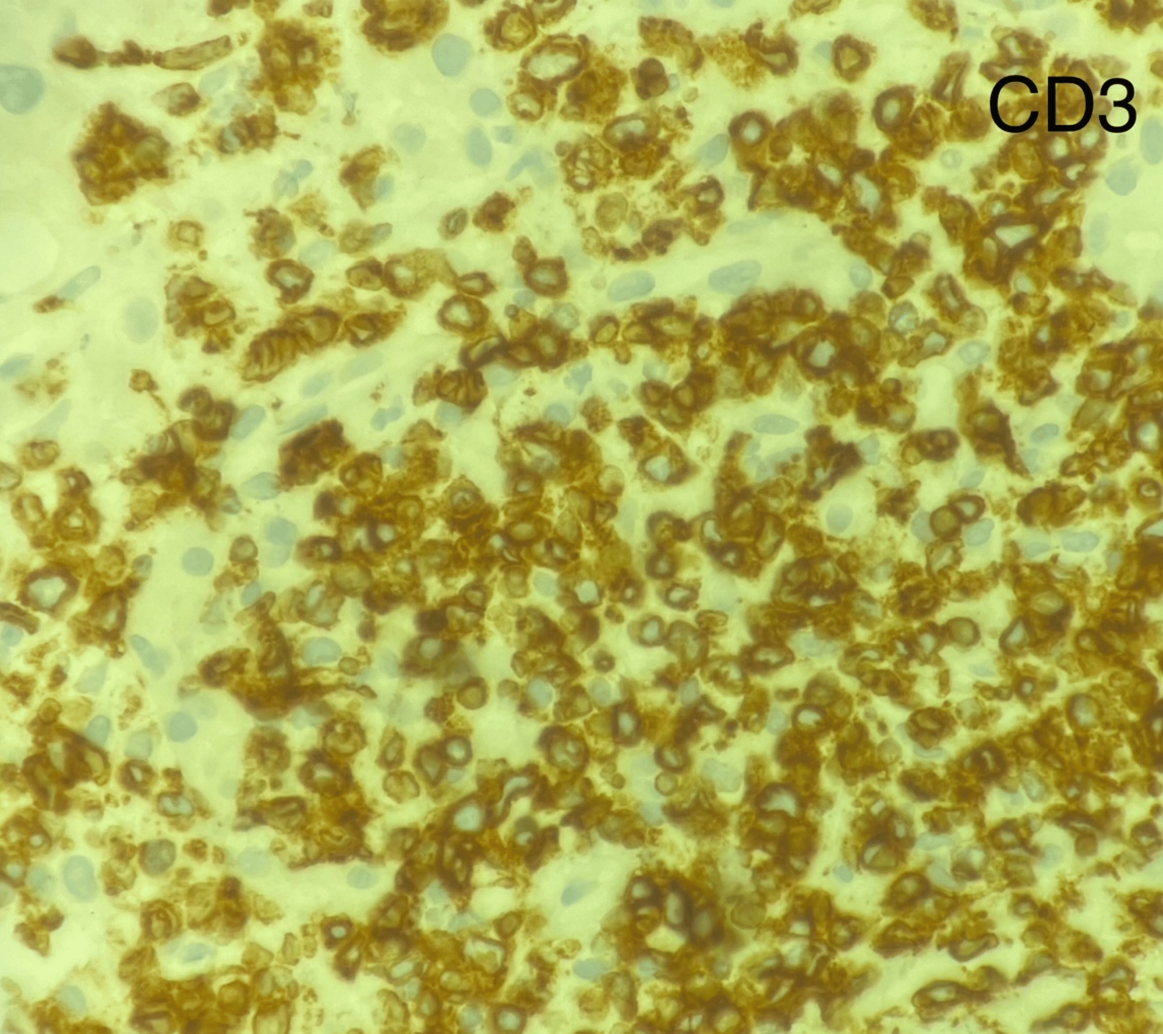
CD3 immunohistochemistry preparation on liver biopsy shows high amounts of T-cells.

**Figure 6:**
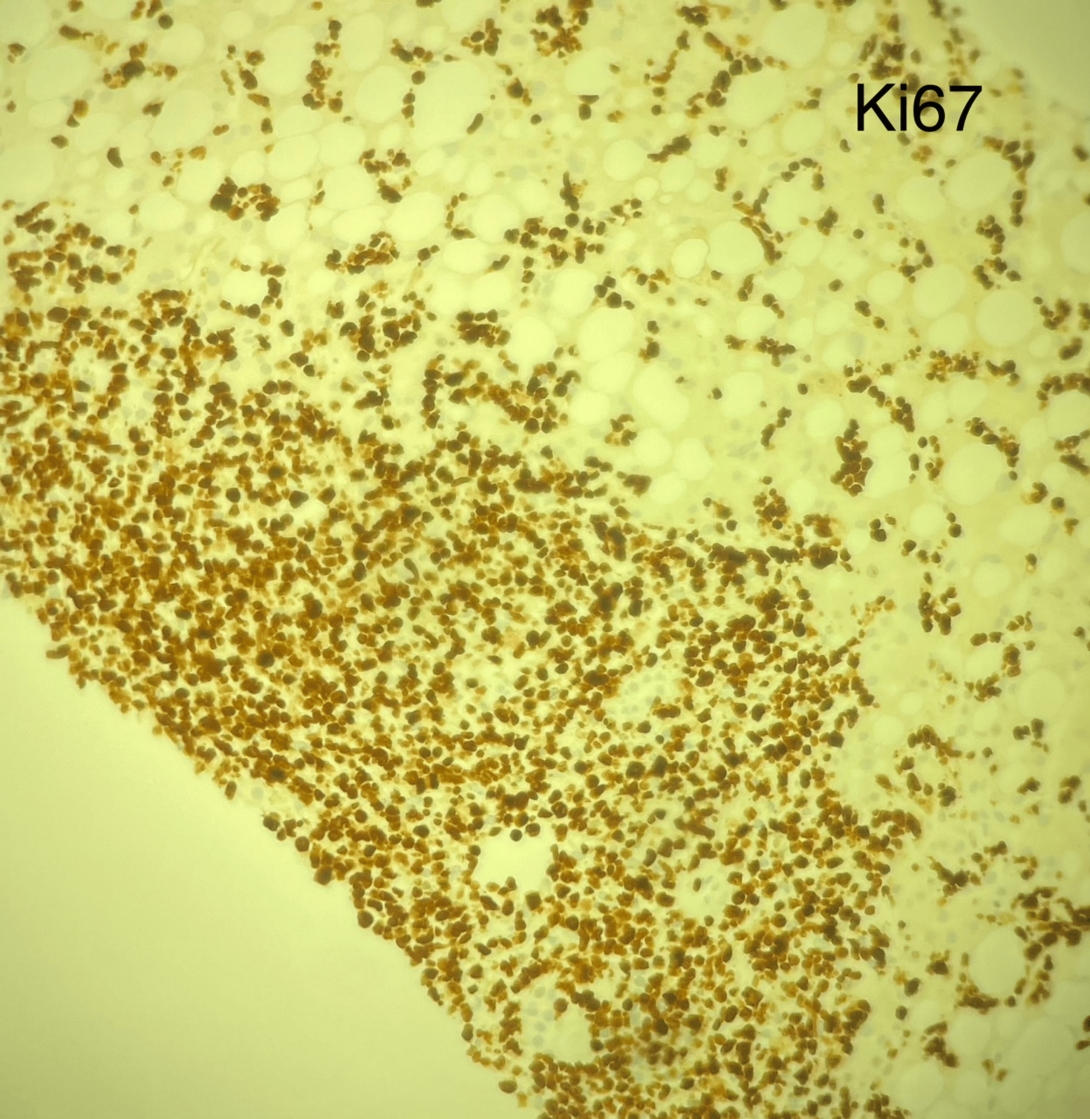
Ki-67 immunohistochemistry preparation on liver biopsy indicates high rates of cellular proliferation.

Several days later, the soluble interleukin-2 receptor alpha (sCD25) levels and the liver biopsy pathology resulted. sCD25 level is a marker of T-cell activation, and an elevated level (>2400 U/mL) is one of the eight criteria used to diagnose HLH. The patient's sCD25 level was 98,110, thus confirming the diagnosis of HLH in this patient by meeting 5/8 HLH-2004 criteria. The liver biopsy specimens were stained with CD20, CD3, and Ki67. Minimal uptake for CD20 and increased cellular uptake for CD3 and Ki67 indicate a peripheral T-cell lymphoma of the liver (Table 2, Figures 3-6) [8]. 

**Table 2:** Other laboratory tests

Other labs	Patient value	Reference range and units
Interleukin 2 receptor (CD25)	98,110	175.3-858.2 pg/ml
Ferritin	13,384	38-380 ng/mL
Lactate dehydrogenase (LDH)	>10,000	84-246 U/L
Cortisol	3.40	Early AM: 6.7-22.6 ug/dL PM: 0.0-10.0 ug/dL
Acetaminophen	<3.0	10.0-30.0 ug/mL
Salicylate	<2.0	5.0-15.0 mg/dL
Creatine kinase	316	39-308 U/L
C3 complement	74	82-185 mg/dL
C4 complement	22	15-53 mg/dL

**Table 3:** Screening tests

Autoimmune tests	Patient value	Reference value
ANCA	Negative	Negative
Anti-dsDNA	Negative	Negative
GBM antibody	Negative	Negative
Infection tests	Patient value	Reference value
T. gondii	Negative	Negative
Rickettsia antibody	Negative	Negative
Rocky mountain spotted fever	Negative	Negative
Adenovirus	Negative	Negative
Beta-D glucan	Positive	Negative
Bartonella	Negative	Negative
Stool culture (Salmonella, Shigella, Shiga toxin, E. coli 0157, Campylobacter)	Negative	Negative
HIV	Negative	Negative
Syphilis	Negative	Negative
COVID	Negative	Negative

## Discussion

HLH syndrome caused by lymphoma is a rare and challenging disease to identify and may be triggered by an idiopathic mechanism (genetic) versus a secondary cause such as malignancy, infection, medication, or autoimmune causes. This patient had a liver biopsy that confirmed a T-cell lymphoma of the liver, which most likely accounted for the patient's drastic presentation and rapid decline. This patient most likely had a peripheral T-cell lymphomas (PTCL), which account for roughly 10% of all non-Hodgkin lymphomas (NHLs). The most common subtype of peripheral T-cell lymphoma (PTCL) is peripheral T-cell lymphoma not otherwise specified (PTCL-NOS), which accounts for nearly 26% of cases, and there is also HSTCL, which accounts for less than 5% [9]. This patient exhibited features of both subtypes of PTCL, but without further testing, it is difficult to accurately identify the specific lymphoma subtype. 

PTCL-NOS may or may not involve nodal sites, but may affect the liver, bone marrow, gastrointestinal tract, or skin, and has worse survival outcomes compared to B-cell lymphomas. PCTL-NOS will typically express CD30, be CD4+/CD8-, and is associated with two major molecular subgroups, GATA3 or TBX21 (NCCN) [9]. HSTCL is characterized by a proliferation of small- to medium-sized mature T cells infiltrating the sinusoids of the liver and spleen. HSTCL cells are typically CD4- and CD8-, may express CD3, CD2, CD7, and CD56, but will be negative for CD5, C1a, and CD10. These neoplastic T cells come from the 𝛾𝛅 T-cell receptor (TCR) expressing lymphocytes, and roughly 20% can express the ⍺β TCR [8]. 

The majority of cases of HSTCL occur de novo, with roughly 20% arising in the setting of immunosuppression, autoimmune disorders such as inflammatory bowel disease, hematologic malignancies, or in those with a history of previous solid organ transplant. HSCLT occurs predominantly in young adults, with the median age of 34 years, and affects men more than women. Patients typically present with constitutional symptoms or just abdominal discomfort due to splenomegaly and or hepatomegaly. Cytopenias are common, and thrombocytopenia may be a sign of disease progression. Hemophagocytic syndrome can also occur and can be associated with a rapid clinical course [8]. Given its rarity and the absence of nodal involvement in most cases, diagnosis of HSTCL can be challenging, and the disease can mimic infectious etiologies or other malignant disorders, resulting in significant delays in diagnosis and initiation of appropriate treatment. Based on this and the rest of the clinical data, we can conclude that the patient most likely had a peripheral T-cell lymphoma (PTCL) of the liver, and the subtype could have been either a PTCL or HSTCL. However, further immunohistochemistry and gene expression profiling are required to accurately classify this lymphoma.

There are significant challenges in rapid lymphoma diagnosis due to the variable presentations of the disease in the hospital. One study found that only serum LDH and lactate levels were the key defining characteristics in differentiating sepsis in a lymphoma patient from sepsis in patients with progressive lymphoma [10]. These abnormal lab findings are attributed to increased cell turnover and tumor hypermetabolism in patients with progressive lymphoma. Many other reports initially work up a patient for septic shock or multi-organ failure before discovering an occult lymphoma many days later. One patient was found to have intravascular lymphoma on postmortem evaluation while initial presentation showed an elevated CRP level, thrombocytopenia, hyperbilirubinemia, renal insufficiency, and shock without a source of infection [11]. Another report details a patient who presented with sub-acute liver failure and jaundice and was initially worked up with suspicion for drug-induced liver injury and alcoholic cirrhosis due to lack of clinical or radiologic evidence of lymphadenopathy or other mass lesions. This patient was ultimately found to have hepatic follicular lymphoma [12].  

Tumor cells are known to change their metabolism over to the glycolytic pathway, which produces lactic acid, also known as the Warburg effect [13]. Most patients do not develop acidosis because of the Cori cycle, but in a few patients, this countermeasure breaks down, and they can develop severe acidosis. Since lactic acid is cleared by the kidneys and liver, if a patient has decreased renal clearance, they have a higher risk of developing type B lactic acidosis. Treatments such as thiamine infusion, hemodialysis, or treatment of the underlying malignancy are all appropriate choices, although the best and fastest option can be difficult to differentiate [14]. Furthermore, when there are multiple etiologies potentially causing the acidosis, accurate diagnosis may not come swiftly.

HLH syndrome can be triggered by lymphoma, and it carries with it a predictor of poor outcomes. HLH is a rare immune activation syndrome where the overproduction of inflammatory cytokines and the inappropriate activation of cytotoxic lymphocytes and macrophages leads to significant morbidity and often eventual mortality [1-5]. These activated cells then infiltrate organs and the nervous system, resulting in seizures, encephalopathy, fever, lymphadenopathy, splenomegaly, hepatomegaly and liver dysfunction, cytopenia, and coagulopathy [6]. HLH patients are also known to develop DIC, which is often the main cause of death [6]. 

This patient met criteria for the HLH syndrome based on splenomegaly, hemoglobinemia, thrombocytopenia, elevated ferritin, elevated triglycerides, and elevated sCD25. Clinicians utilize the Histiocyte Society’s HLH-94 or 2004 diagnostic criteria which includes either positive HLH molecular diagnosis or five out of eight of the following criteria: fever (>38.5 C); hypertriglyceridemia (fasting triglycerides >/3.0 mmol/L) and/or hypofibrogenemia (fibrinogen <1.50 g/L); cytopenia affecting 2>/ of three cell types: hemoglobin <90 g/L (in infants <4 weeks: hemoglobin <100 g/L), platelets <100 x 109/L, neutrophils (1.0 x 109/L), splenomegaly, and hemophagocytosis in the lymph nodes, spleen, and bone marrow; and new criteria including ferritin >/ 500 mg/L, low or absent NK-cell activity, and soluble CD25 >/2400 U/mL [7]. Certain clinicians prefer ferritin >3000 ng/mL as this may be more diagnostic for HLH. In addition to blood tests, patients may undergo cross-sectional imaging studies such as CT or MRI, lumbar puncture, and/or have bone marrow aspirate and biopsy to confirm hemophagocytosis.  

Although previously categorized as primary versus secondary HLH, the North American Consortium for Histiocytosis (NACHO) now recommends categorizing HLH by common precipitating factors or associated diseases [2]. HLH is now classified as familial HLH (F-HLH), HLH associated with malignancy (M-HLH), lymphoma-associated hemophagocytic syndrome (LAHS), natural killer/T-cell LAHS, HLH occurring with certain immune-activating therapies or treatment-related (Rx-HLH), HLH occurring with immune compromise, either primary or acquired (IC-HLH), HLH associated with rheumatic conditions (Rh-HLH), and HLH not associated with other specific conditions (HLH-NOS) [6]. F-HLH can occur due to several gene mutations, including those of the perforin gene (PRF1, UNC13D, STX11) or those related to T-cell function (SH2D1A, ITK, CD27) [1, 15-16]. Secondary or non-familial HLH can also be triggered by immune activation, such as bacterial or viral infections (e.g., cytomegalovirus, Epstein-Barr Virus, parvovirus, and adenovirus).   

Compared to other HLH subtypes, LAHS is often misdiagnosed but is rapid in onset and progression, with an overall poor prognosis due to its high early mortality rate [17]. LAHS is the most common secondary hemophagocytic syndrome in the United States, although natural killer (NK)/T-cell lymphoma HS and T-cell lymphoma HS are not as frequently diagnosed in the United States when compared to Asian countries [18]. Patients with T-LAHS are also more prone to DIC when compared to B-cell LAHs patients [6-7].  

A study of Asian patients showed that the median survival time for LAH patients overall was 43 days, 55 days for B-cell LAHs, and 40 days for NK/T-LAHS [19]. Another study found that patients with NK/TLAHS had a mortality rate of 96.4% and a 15-day median survival time [20]. Patients with LAH who do not get chemotherapy have a median survival time of 11 days [21].  

HLH is more common in males, typically in their 50s, while F-HLH is more common in pediatric patients [22-23]. HLH patients most often present with a sudden onset fever, multiple organ failure, hypotension requiring vasopressors, and respiratory failure requiring mechanical ventilation. In a study of 139 cases based in Asia, these patients also had hepatomegaly and lymphadenopathy when compared to non-LAHS patients [17].  

Due to its various precipitating factors, there is no standard treatment regimen for HLH. Most practitioners use the HPS-94 or HPS-2004 treatment protocols as a guide in addition to utilizing steroids, intravenous immunoglobulin, CD25 monoclonal antibody treatment, and multiple lymphoma-specific treatments that will be described in detail below [18]. Patients may also receive hematopoietic stem cell transplantation if diagnosed with F-HLH.  

The treatment of LAHS begins with glucocorticoid treatment followed by etoposide-based treatments or CHOP protocol (cyclophosphamide, hydroxydaunorubicin, oncovin, and prednisone) [7]. There was a study that utilized the COPE or ECHOP regimen (etoposide, dexamethasone, vindesine, cyclophosphamide, and nordoxorubicin) for NK/T-cell lymphoma or T-cell lymphoma, while the R-CHOP regimen (rituximab, dexamethasone, vindesine, cyclophosphamide, and nordoxorubicin) was used for non-Hodgkin’s B-cell lymphoma, and the ABVD regimen (epirubicin, bleomycin, vindesin, and dacarbazine) for Hodgkin’s lymphoma. Mortality rate was still 50.4% among these patients despite treatment [17].   

## Conclusions

This patient presented with complaints of abdominal pain, nausea, and vomiting and was found to have severe lactic acidosis and renal failure that rapidly progressed to multi-organ failure, leading to a severe coagulopathy and internal bleeding, which became fatal. It became clear that this patient was not suffering from an intra-abdominal infection causing organ dysfunction but rather a rapidly progressive HLH syndrome. The inciting cause for the HLH was not clear, but given the negative infectious workup and rapid decline, malignancy was suspected. The liver biopsy with the diagnosis of T-cell lymphoma was performed several days after the case study patient’s death. T-cell lymphoma of the liver is exceedingly rare compared to B-cell lymphomas and is associated with worse outcomes. Based on the available data, a hepatosplenic T-cell lymphoma is the most likely subtype of lymphoma, as it invades the liver sinusoids, does not have extensive nodal involvement, and is associated with HLH syndrome. It is unclear if earlier diagnosis would have changed management, given the patient’s rapid clinical deterioration in spite of maximal medical therapy. This unique case of peripheral T-cell lymphoma highlights the need for early diagnosis of malignancy to better treat patients presenting with rapid multi-organ failure of unclear etiology. 
